# Predicting the Impacts of Climate Change on the Potential Distribution of Major Native Non-Food Bioenergy Plants in China

**DOI:** 10.1371/journal.pone.0111587

**Published:** 2014-11-03

**Authors:** Wenguo Wang, Xiaoyu Tang, Qili Zhu, Ke Pan, Qichun Hu, Mingxiong He, Jiatang Li

**Affiliations:** 1 Key Laboratory of Development and Application of Rural Renewable Energy, Biogas Institute of Ministry of Agriculture, Chengdu, 610064, P.R. China; 2 Chengdu Institute of Biology, Chinese Academy of Sciences, Chengdu, 610064, P.R. China; University of California Davis, United States of America

## Abstract

Planting non-food bioenergy crops on marginal lands is an alternative bioenergy development solution in China. Native non-food bioenergy plants are also considered to be a wise choice to reduce the threat of invasive plants. In this study, the impacts of climate change (a consensus of IPCC scenarios A2a for 2080) on the potential distribution of nine non-food bioenergy plants native to China (*viz.*, *Pistacia chinensis*, *Cornus wilsoniana*, *Xanthoceras sorbifolia*, *Vernicia fordii*, *Sapium sebiferum*, *Miscanthus sinensis*, *M. floridulus*, *M. sacchariflorus* and *Arundo donax*) were analyzed using a MaxEnt species distribution model. The suitable habitats of the nine non-food plants were distributed in the regions east of the Mongolian Plateau and the Tibetan Plateau, where the arable land is primarily used for food production. Thus, the large-scale cultivation of those plants for energy production will have to rely on the marginal lands. The variables of “precipitation of the warmest quarter” and “annual mean temperature” were the most important bioclimatic variables for most of the nine plants according to the MaxEnt modeling results. Global warming in coming decades may result in a decrease in the extent of suitable habitat in the tropics but will have little effect on the total distribution area of each plant. The results indicated that it will be possible to grow these plants on marginal lands within these areas in the future. This work should be beneficial for the domestication and cultivation of those bioenergy plants and should facilitate land-use planning for bioenergy crops in China.

## Introduction

The declining availability of fossil fuels in a world of growing population and the environmental impact of greenhouse gas emissions have motivated increasing interest in the production of renewable bioenergy [1]–[3], which may become a substantial proportion of our future energy supply [4]. Bioenergy crops are the most important raw materials used for producing bioenergy, especially for liquid biofuel [5]. In recent decades, a greater number of wild plant species have been cultivated for bioenergy production, and some easily adaptable species have been introduced to different regions and countries. For example, an American native plant, switchgrass (*Panicum virgatum*), has been introduced to many other parts of the world, including China [6] and Europe [7]. For some regions, the introduction of alien species with high primary productivity can be beneficial for bioenergy production; however, the presence of such plants may be detrimental to the regional ecosystems [8]. For example, the giant reed (*Arundo donax*) is among the species with the highest biofuel potential in Europe [9], [10] but has also been named one of the world's top 100 worst invaders. Being aware of the seriousness of this problem, some researchers have conducted assessments of the invasive potential of some bioenergy plants in some countries and regions, demonstrating the high invasive probability of some species [11]–[13]. Thus, more attention has been paid in recent years to native bioenergy plants [14]–[16].

As the world's largest energy consumer [17], China is also paying more and more attention to bioenergy [18], [19], with particular focus on energy produced from non-food bioenergy crops. These crops are mainly grown on marginal lands, *i.e.*, land in relatively poor natural condition that remains capable of supporting the cultivation of energy plants, or land that is not currently used for agricultural production but can grow energy plants [20]. Because it is necessary to ensure the security of the food supply, there is almost no currently cultivated land that could be made available to grow bioenergy crops in China [20]–[22]. Hence, several bioenergy plants have been introduced to China, and their use has been explored over the last few years. However, some alien species such as *Helianthus tuberosus* and *Ricinus communis* also showed the potential for biological invasion [23]. On the other hand, China is one of the world's most abundant countries in terms of plant resources, especially in energy plant resources [22], [24]. Although many wild or semi-wild native non-food bioenergy plant species have been gradually cultivated [25], [26], there is still a long way to go before cultivation of bioenergy feedstocks can occur on a large scale.

To further develop native bioenergy feedstock crops, it is necessary to understand the climate niche of those plants and identify their suitable growing areas under current and future climate conditions. Species distribution models (SDMs), which predict species' probability of occurrence across a landscape by relating documented locations of their presence to environmental information, are frequently used to solve this problem [27], [28]. Many SDMs, such as Genetic Algorithm for Rule-set Production (GARP), Ecological Niche Factor Analysis (ENFA), Maximum Entropy (MaxEnt) and BIOCLIM have been developed for estimating species distributions from presence-only species records. Among them, MaxEnt usually produces good predictions of species distribution [29]–[34]. MaxEnt is a popularly used SDM for modeling species distributions. Its predictive applications include the spatial distribution of plant species [35], the spread of invasive species [36], and the effects of climate change on plant distributions [37], [38]. The model has also been applied to identify the current and future suitable areas for growing bioenergy crops [39], [40]. In this study, the MaxEnt model was used to analyze the climatic niche as well as the present and future potential distributions of nine native non-food plants with great potential for bioenergy production in China. This knowledge will be of fundamental importance for further domestication and cultivation of those plants, and for the planning, implementation, and operational management of future bioenergy production both in China and around the world.

## Materials and Methods

### Study area and climate

China covers approximately 9.6 million square kilometers of land area, but the agricultural crop land area is only 1.4 million square kilometers. Most parts of China lie in the North Temperate Zone, which is characterized by a warm climate and distinctive seasons. Thus, a continental monsoon climate prevails over most of China. From September to April the following year, the dry and cold winter monsoons result in cold and dry winters and lead to large differences in temperature between the North and the South. From April to September, warm and humid summer monsoons give rise to high temperatures and abundant rainfall all over China and lead to small differences in temperature between the North and the South. In terms of temperature, the country can be divided into six zones; these are the tropical, subtropical, warm-temperate, mid-temperate,and cold-temperate zones from south to north; a plateau climate zone in the Tibetan Plateau (Figure S1, S2). There is a decreasing trend in precipitation from the southeastern coastal to the northwestern inland area, and the average annual precipitation differs significantly from place to place (Figure S3).

### Study species

Nine native non-food bioenergy plants, including five woody oil plants and four grasses, were selected for this study (Table 1). The oil plants were *Pistacia chinensis*, *Cornus wilsoniana*, *Xanthoceras sorbifolia*, *Vernicia fordii* and *Sapium sebiferum*; these deciduous trees or shrubs were traditionally used for timber, landscaping and technical oils, etc. [41]–[46]. The grasses consisted of *Miscanthus sinensis*, *M. floridulus*, *M. sacchariflorus* and *Arundo donax*. These species are traditionally used for forage and papermaking as well as water and soil conservation in China [41], [47], [48]. However, in North America and Europe, they are planted as energy crops to produce cellulosic ethanol [9], [49].

**Table 1:** Nine native non-food bioenergy plants distributed in China.

Species	Plant Family	Growth habit	Biomass type	Natural habitats and altitude distribution	Traditional use	Number of herbarium records collected in this study
*Pistacia chinensis*	Anacardiaceae	Deciduous trees	Fatty acids from seed oil	Hills and mountain forests on rocky soils; 100–3600 m.	Timber, landscaping, yellow dye	358
*Cornus wilsoniana*	Cornaceae	Deciduous trees	Fatty acids from Fruit oil	Forests; 100–1100 m.	Timber, landscaping, livestock feed	112
*Xanthoceras sorbifolia*	Sapindaceae	Deciduous shrubs or small trees	Fatty acids from seed kernels oil	Hills and slopes, 52–2260 m	Timber, landscaping, edible	155
*Vernicia fordii*	Euphorbiaceae	Deciduous trees	Fatty acids from seed kernel oil	Open forests; 200–1500(-2000) m, usually cultivated on slopes below 800 m.	Technical oils	620
*Sapium sebiferum*	Euphorbiaceae	Deciduous trees	Fatty acids from seed oil	open field, open forests, widely cultivated	Technical oils, landscaping	309
*Miscanthus sinensis*	Poaceae	Perennial herb	Cellulose	Mountain slopes, coasts, disturbed places; below 2000 m.	Forage, papermaking	260
*Miscanthus floridulus*	Poaceae	Perennial herb	Cellulose	Slopes, valleys, grassy places.	Forage, papermaking, ornamentals	359
*Miscanthus sacchariflorus*	Poaceae	Perennial herb	Cellulose	Mountain slopes, river banks	water and soil conservation	228
*Arundo donax*	Poaceae	Perennial herb	Cellulose	River banks and other damp places, but it will also grow when planted in semiarid habitats.	Forage, papermaking, ornaments	518

### Species data collection

The occurrence records of the species of the nine plants were collected from on-line herbarium specimen information provided by the Chinese Virtual Herbarium (http://www.cvh.org.cn/cms/en), the Specimen Resources Sharing Platform for Education (http://mnh.scu.edu.cn/new/) and the Global Biodiversity Information Facility (www.gbif.org). Between 112 and 620 herbarium records were collected from each species (Table 1).

### Climatic data

Present climatic data (1950–2000) were downloaded from the WorldClim database with 2.5-min resolution (http://www.worldclim.org/) [50]. Climatic data projected to the year 2080 from the global climate model of the Canadian Centre for Climate Modeling and Analysis (CCCMA) were used to assess the effects of climate change. The CCCMA model was recently evaluated as a top-performing model [51]. The greenhouse gas emission scenario A2a was selected to assess plausible futures based on a range of human choices over the next few decades. Nineteen bioclimatic variables from the WorldClim dataset were used to assess current climatic conditions (Table 2).

**Table 2:** Percent contributions of the bioclimatic variables in the MaxEnt models for the nine target bioenergy plants.

Environmental variables (Unit)	Percent contribution								
	*Pistacia chinensis*	*Cornus wilsoniana*	*Xanthoceras sorbifolia*	*Vernicia fordii*	*Sapium sebiferum*	*Miscanthus sinensis*	*Miscanthus floridulus*	*Miscanthus sacchariflorus*	*Arundo donax*
Bio1 Annual mean temperature (°C)	10.7	1.1	20.0	14.2	21.7	1.3	7.4	1.1	15.1
Bio2 Mean diurnal range (mean of monthly max. and min. temp.) (°C)	2.9	7.1	1.4	0.9	0.3	0.4	8.1	0.1	1.7
Bio3 Isothermality (Bio2/Bio7) ×100) (–)	18.1	5.7	0.1	13.2	21.4	22.1	1.1	0.0	2.5
Bio4 Temperature seasonality (standard deviation ×100) (C of V)	0.8	15.6	7.3	0.9	2.2	0.3	1.3	19.0	11.7
Bio5 Maximum temperature of warmest month (°C)	0.8	0.3	0.2	0.7	0.0	0.6	0.0	0.8	1.7
Bio6 Minimum temperature of coldest month (°C)	0.4	2.4	0.3	7.5	0.0	3.5	0.1	0.0	0.2
Bio7 Temperature annual range (Bio5–Bio6) (°C)	1.1	0.1	5.3	1.1	0.8	0.1	0.8	0.7	0.2
Bio8 Mean temperature of wettest quarter (°C)	7.0	0.1	4.9	0.2	0.1	7.0	0.2	9.4	1.0
Bio9 Mean temperature of driest quarter (°C)	2.8	7.9	13.5	0.0	0.1	6.6	12.0	12.3	2.6
Bio10 Mean temperature of warmest quarter (°C)	0.2	0.6	0.1	1.6	1.9	7.0	0.1	11.6	0.5
Bio11 Mean temperature of coldest quarter (°C)	4.8	6.9	10.2	8.0	2.8	0.0	2.2	1.4	39.0
Bio12 Annual precipitation (mm)	0.1	0.1	1.1	0.1	17.3	1.0	3.6	1.8	6.8
Bio13 Precipitation of wettest period (mm)	0.1	0.0	2.4	0.1	3.7	0.3	0.2	12.2	0.8
Bio14 Precipitation of driest period (mm)	0.2	0.0	0.0	0.6	2.1	0.5	2.6	0.3	0.2
Bio15 Precipitation seasonality (CV) (C of V)	2.9	4.4	13.0	0.1	0.2	4.3	1.0	3.4	0.8
Bio16 Precipitation of wettest quarter (mm)	0.0	0.0	0.1	14.3	0.1	7.7	0.1	0.5	7.9
Bio17 Precipitation of driest quarter (mm)	0.1	0.0	0.1	0.0	0.0	0.1	0.0	0.0	0.0
Bio18 Precipitation of warmest quarter (mm)	46.3	45.5	9.6	36.4	25.1	36.5	59.0	25.1	0.4
Bio19 Precipitation of coldest quarter (mm)	0.5	2.2	10.4	0.1	0.1	0.7	0.1	0.4	6.8

### Maximum entropy algorithm (MaxEnt)

A maximum entropy (MaxEnt version 3.3.3k; http://www.cs.princeton.edu/wschapire/maxent/) approach was employed to model present and future potential distributions of these nine species [33]. The 25th percentile training presence, a convergence threshold of 10^−5^, a maximum of 500 iterations and 10,000 global background points were used. The logistic output was chosen as an estimate of the probability of the presence (ranging from 0 to 1) conditioned on the environmental variables in each grid cell [52], given that the temporal and spatial scale of sampling results in a 50% chance of the species being present in suitable areas [34].

To predict the distribution of each plant, MaxEnt's internal jackknife procedure and the contribution percentage of each variable were used to assess the importance of each environmental variable. Contribution percentage was gauged the gain in model performance with and without each variable, essentially providing a measure of the relative importance of each environmental variable. For all predictions, models were evaluated by the area under receiver operating curve (ROC) statistic, which is used to calculate the area under the ROC curve (AUC) based on the trapezoidal method described in [33]. The AUC values were calculated automatically by MaxEnt. During prediction process, the false-positive error rate on the x-axis versus the true positive rate along the y-axis for every probability value predicted by the model was plotted in a coordinate system. The AUC value is the sum of the area occurring under the ROC curve and varies from 0 to 1. Generally, an AUC value of 0.5 indicates that model did not perform better than random, values between 0.5 and 0.75 are indicative of low model performance, values of 0.75–0.9 are considered to be potentially useful, and values above 0.9 are excellent [53], [54].

## Results

### Model accuracy and prediction success

Models for the nine species performed better than random. Both training and test AUC values were greater than 0.9 for all species under the present and future climatic conditions (Table 3). These modeling results were considered to be of an excellent standard. These results indicated that for each species, the most climatically suitable areas predicted by MaxEnt were highly correlated with the occurrence of location points.

**Table 3:** The area under receiver operating curve (AUC) score of MaxEnt models for each of the nine bioenergy plants.

Species	Current		Future (2080)	
	Test AUC	Training AUC	Test AUC	Training AUC
*Pistacia chinensis*	0.974	0.978	0.974	0.979
*Cornus wilsoniana*	0.987	0.993	0.986	0.993
*Xanthoceras sorbifolia*	0.987	0.989	0.989	0.990
*Vernicia fordii*	0.976	0.981	0.979	0.981
*Sapium sebiferum*	0.963	0.968	0.964	0.970
*Miscanthus sinensis*	0.973	0.979	0.964	0.979
*Miscanthus floridulus*	0.977	0.979	0.978	0.980
*Miscanthus sacchariflorus*	0.983	0.987	0.987	0.988
*Arundo donax*	0.927	0.947	0.936	0.942

### Projections suitable habitats under baseline climate (1950–2000)

**Figure 1:**
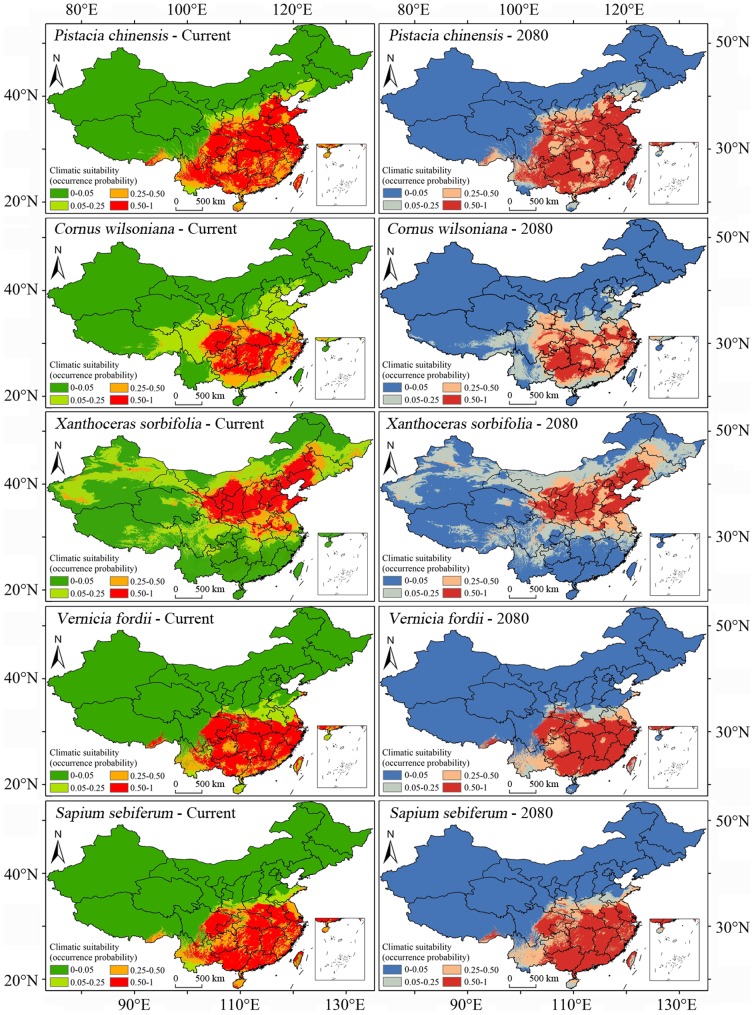
Predicted current and future (2080) suitable habitats for five woody oil plants (*Pistacia chinensis*, *Cornus wilsoniana*, *Xanthoceras sorbifolia*, *Vernicia fordii* and *Sapium sebiferum*).

Figure 1 shows the suitable habitats predicted by MaxEnt for the five oil bioenergy plants in China. The main suitable habitats of the five plants, dominated by hills and plains, are distributed in the regions east of the Mongolian Plateau and the Tibetan Plateau. These plants are wildly or semi-wildly distributed on hills or slopes and in mountain forests, open forests or open field (Table 1). With the exception of *X. sorbifolia*, suitable areas for the plants are mainly found in the south of China, including the Yangtze Plain, the Southeast China Hill, the Sichuan Basin and the Yunnan-Guizhou Plateau. In contrast, the suitable habitats of *X. sorbifolia* are distributed in the north of China, including the Northeast China Plain, the North China Plain and the Loess Plateau. *P. chinensis*, *V. fordii* and *S. sebiferum* are adapted to some parts of the tropics, while the other two oil trees were distributed in the temperate and subtropical regions. Among these five plants, *P. chinensis* has the widest suitable habitat according to the MaxEnt model's results.

**Figure 2:**
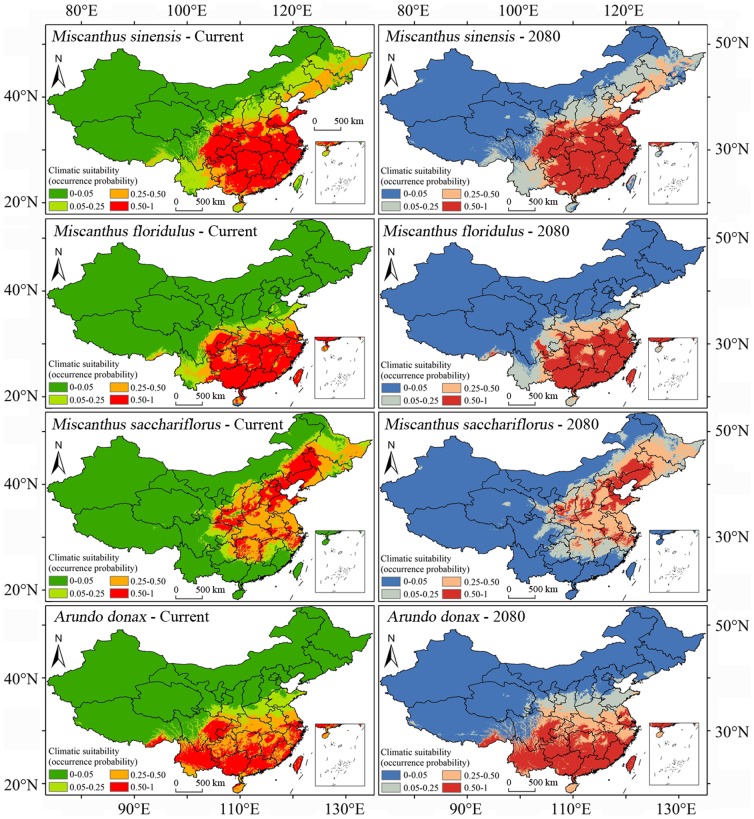
Predicted current and future (2080) suitable habitats for four bioenergy grasses (*Miscanthus sinensis*, *M. floridulus*, *M. sacchariflorus* and *Arundo donax*).

The plants in the genus *Miscanthus* are famous for their great potential for second-generation bioethanol production [49]. There are a total of 14 species in this genus, which originated from Southeast Asia and the Pacific Islands, even extending to tropical Africa, and seven species of them are distributed in different regions of China [47]. In China, *M. sinensis*, *M. floridulus* and *M. sacchariflorus* are the most widely distributed species in the genus. Figure 2 showed the suitable habitats for these three species by MaxEnt prediction. *M. sinensis* and *M. floridulus* are mainly distributed in the south, the tropical and subtropical regions. The suitable area of *M. sacchariflorus* ranges from the Songhua River Basin in the northeast to the Yangtze River Basin in the south. *A. donax* is also a native grass in China, and its suitable habitats include the Sichuan Basin, the Yunnan-Guizhou Plateau and the area south of the Yangtze River.

### Dominant bioclimatic variable analysis

In this study, bioclimatic variables were chosen as predictors for the potential distribution of the nine species. Table 2 shows the contribution percentage of the 19 bioclimatic variables to each species. The distributions of *P. chinensis*, *C. wilsoniana*, *V. fordii*, *M. sinensis* and *M. floridulus* were significantly affected by precipitation, especially the precipitation of the warmest quarter (Bio 18), the contribution percentages of which were more than 35% for all the five plants and approaching 59% for *M. floridulus*. However, the distribution of *X. sorbifolia* was mainly predicted by the temperature, especially the annual mean temperature (Bio 1, 20%) and the distribution of *A. donax* was predicted by the mean temperature of the coldest quarter (Bio 11, 39%). The precipitation of the warmest quarter (Bio 18, 25.1%) and temperature seasonality (Bio 4, 19%) provided the most useful information for *M. sacchariflorus*. Lastly, for *S. sebiferum*, the contributions of precipitation of the warmest quarter (Bio 18, 25.1%), annual mean temperature (Bio 1, 21.7%) and isothermality (Bio 3, 21.4%) were almost the same.

**Figure 3:**
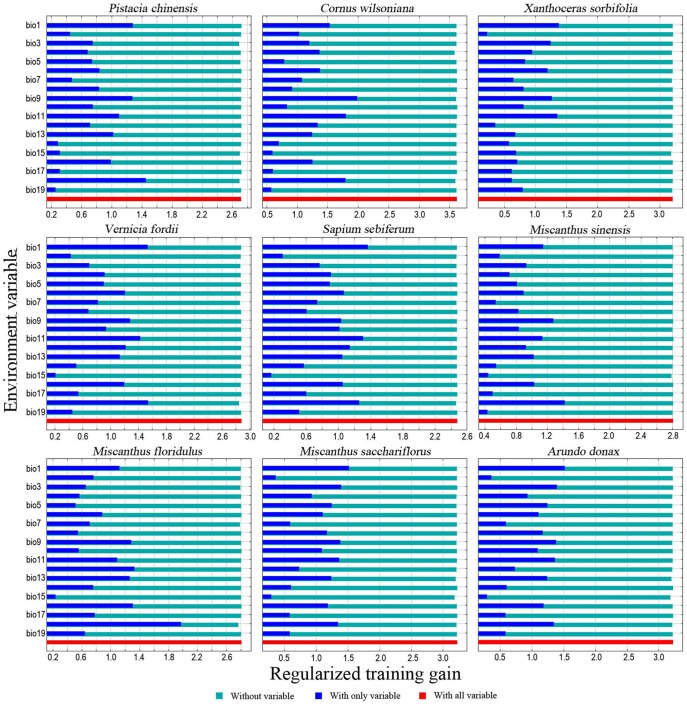
Relative predictive power of different bioclimatic variables based on the jackknife of regularized training gain in MaxEnt models for the nine plants.

The jackknife evaluation procedure indicated that the climatic variable of precipitation of the warmest quarter (Bio 18) was the strongest predictor for the geographic distribution prediction of *P. chinensis*, *V. fordii*, *M. sinensis* and *M. floridulus*. However, for *X. sorbifolia*, *S. sebiferum*, *M. sacchariflorus* and *A. donax*, the most important predictor was the variable of annual mean temperature (Bio 1). The precipitation of the coldest quarter (Bio 19) was the most important variable for *C. wilsoniana* (Figure 3).

### Changes in climatically suitable habitats by 2080

There were subtle changes between current and future suitable habitats predicted for each species (Figure 1, 2). The general trend was that the predicted suitable area in the tropics was decreasing. Taking Hainan Island as an example, some parts were suitable for *P. chinensis*, *S. sebiferum*, *M. floridulus* and *A. donax* to grow under the present climatic conditions. However, for the predicted climatic conditions in 2080 (A2a scenario), there was almost no site with probability greater than 0.5 in the island for all those plants.

**Figure 4:**
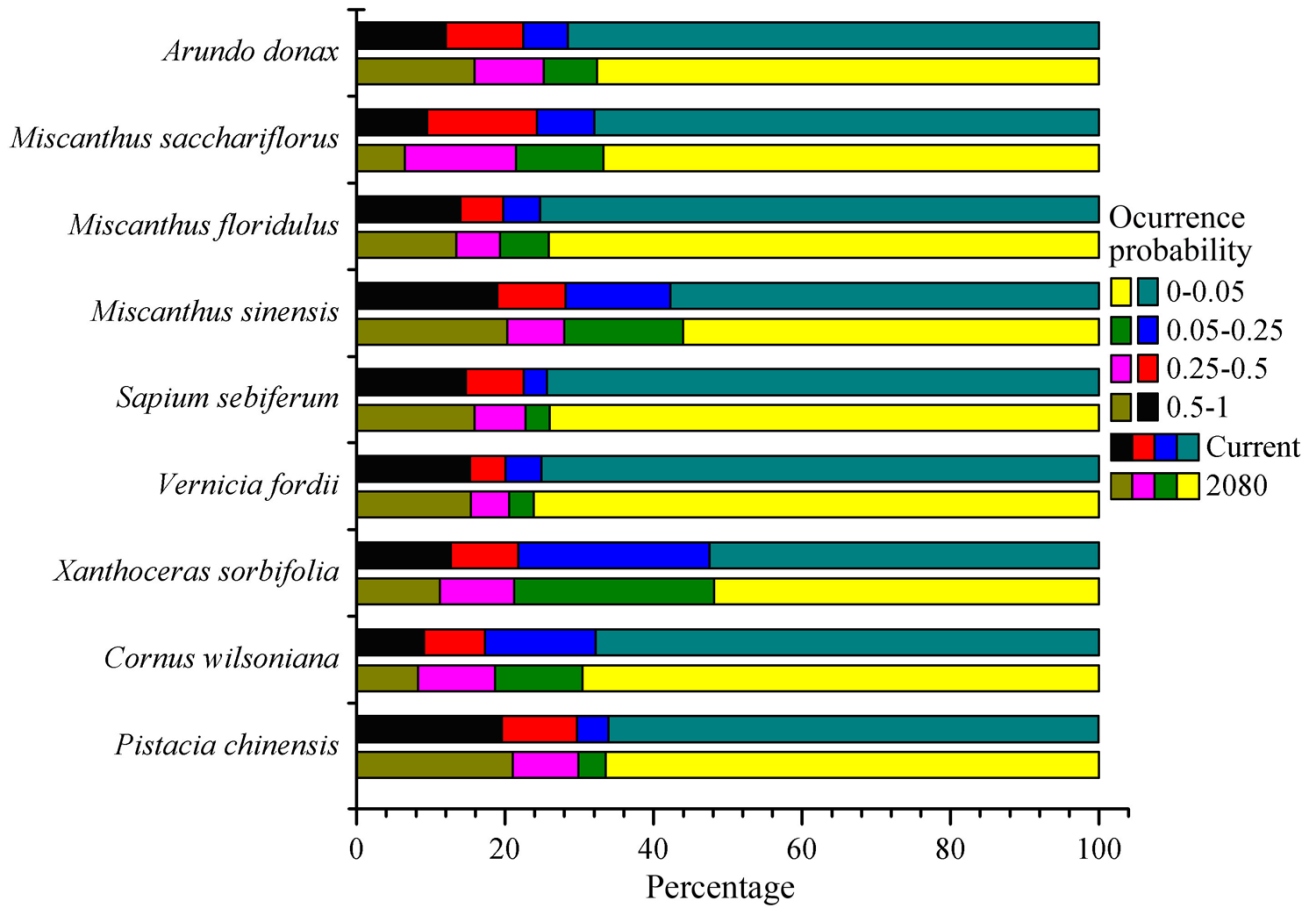
Percentage changes in extent of distributions of the nine bioenergy plants in China under current and future (2080) climatic conditions.

Among the nine species, the extent of suitable habitats (occurrence probability above 0.5) of *P. chinensis*, *S. sebiferum*, *M. sinensis* and *A. donax* would slightly increase under the 2080 climatic conditions, and the same would happen to *V. fordii*. However, the extents for the other four species would slightly decrease (Figure 4).

## Discussion

MaxEnt is an excellent species distribution modeling tool that has been extensively utilized for more and more species distributions predictions since it became available in 2004 [34]. It can model the species distributions just from presence-only species records, which are usually collected from specimen records, field survey records and literatures. The obtained records numbers are various for different species. In this study, up to 620 records were found for *V. fordii*, but only 112 records for *C. wilsoniana* were collected. In some other studies, this number is even lower [37]. However, the MaxEnt model is strongly influenced by species' ecological characteristics independent of sample size and can extract useful biogeographical information from small samples (as low as five records) [55], [56]. The records numbers of the nine species varied widely (in the range of 112–620), but the AUC values were all greater than 0.9, indicating that the predicted results of the tested nine plants were reliable.

MaxEnt provides species' probabilities of occurrence in a logistic output format, which ranges from 0 to 1 assigned to each pixel in the study area [33]. Usually, the values are re-divided into several classes of potential habitats. However, in different reports for different species, the classification approaches were also different. For instance, for *Justicia adhatoda*, the area could be considered as suitable habitat only if the occurrence probability was greater than 0.6 [35], while for *Jatropha curcas*, the occurrence probability can be just above 0.25 [40]. Elith et al. considered that the probability of species being present in suitable areas was at an average site of 0.5 [34]. For each of the nine species in this study, the area with probabilities above 0.5 was the main natural distribution area [41]–[48]. Thus, in this study, we focus on the suitable habitats with probabilities above 0.5.

The current suitable areas of these nine plants were mainly distributed in the regions east of the Mongolian Plateau and Tibetan Plateau, which are the traditional Chinese agricultural regions [57]. The cultivation of these plants relies on marginal land. In China, the total area of marginal land suitable for the cultivation of energy plants is estimated to be up to 130.34 million ha [20]. According to the distribution map of marginal land suitable for energy plants in China [20], except for sparse grasslands, most of the marginal land lies within the suitable areas of the nine plants in this study (Figure 2, 3). The shrub land and sparse forest land should be suitable for the woody oil plants, while the dense grassland, moderate dense grassland and some bottom land or bare land should be appropriate for the energy grasses [20], [22], [24].

Temperature and precipitation are two key factors influencing plant growth and distribution [58]. The suitable area of the nine plants was mostly distributed in humid and semi-humid regions of China, and five of them were mainly distributed in the south of China, which is dominated by humid, subtropical and tropical regions. The precipitation of the warmest quarter (Bio 18) is one of the most important bioclimatic variables. It profoundly affected the distribution of *P. chinensis*, *C. wilsoniana*, *V. fordii*, *M. sinensis* and *M. floridulus*, which were mainly distributed in southern China. For the species distributed in northern China, such as *X. sorbifolia* and *M. sacchariflorus*, some temperature variables such as annual mean temperature (Bio 1), temperature seasonality (Bio 4) and mean temperature of driest quarter (Bio9) made more contribution to their distribution.

Climate change will cause plants' ranges to shift because climate is the dominant factor affecting the natural distribution of plants [59], [60]. The relatively stable distribution region of energy plants is very important for the sustainable supply of feedstock for bioenergy production [39], [40]. Based on recent observations suggesting that climate change will be more severe than previously expected [61], [62], this study predicted energy plant distributions under the A2a emission scenario, which projects relatively large changes. According to the MaxEnt modeling results under this scenario, all nine species would have relatively stable suitable ranges in the following decades (Figure 1, 2); even though some species would suffer small decreases in their range (Figure 3), the impact of climate change on the total suitable area of each plant in the coming decades will likely be limited. However, it is acknowledged that continued global warming will inevitably influence plant growth and distribution [63], [64].

Our result also showed that the suitable habitats of *P. chinensis*, *S. sebiferum*, *M. floridulus* and *A. donax* will decrease in low-latitude regions in the near future. This is similar to the patterns for bioenergy crops in Europe predicted by Tucka et al. [65]. The low-latitude zones are particularly vulnerable to climate change. If bioenergy plants are to be viable in these regions of China in the future, adaptation to climate change will involve efforts to breed or find heat- and drought-tolerant plant varieties or species.

In conclusion, for the nine non-food energy plants selected in this study, the suitable habitats were mainly distributed in the traditional farming areas of China, where arable lands that can be used for the planting of energy plants are rare. The large-scale cultivation of these energy plants in the future can only be conducted on marginal land. Climate change in coming decades will cause decreases in the suitable habitats in the tropics but will have little effect on the total distribution area of each plant, indicating that distribution of those plants in the coming decades will remain relatively stable. Thus, suitable marginal land is available for cultivating those plants. Nevertheless, for bioenergy production to be sustainable, further work, including breeding and cultivation management, still needs to be performed.

## Supporting Information

Figure S1 Regions included in the present study.
(TIF)

Figure S2 Climate zones in China.
(TIF)

Figure S3 Distribution of humid and arid areas in China.
(TIF)
