# Supplementary figures and images for: Predicting the Impacts of Climate Change on the Potential Distribution of Major Native Non-Food Bioenergy Plants in China
Source: PLoS One. 2014 Nov 3;9(11):e111587. doi: 10.1371/journal.pone.0111587 (PMC4218772; doi:10.1371/journal.pone.0111587)

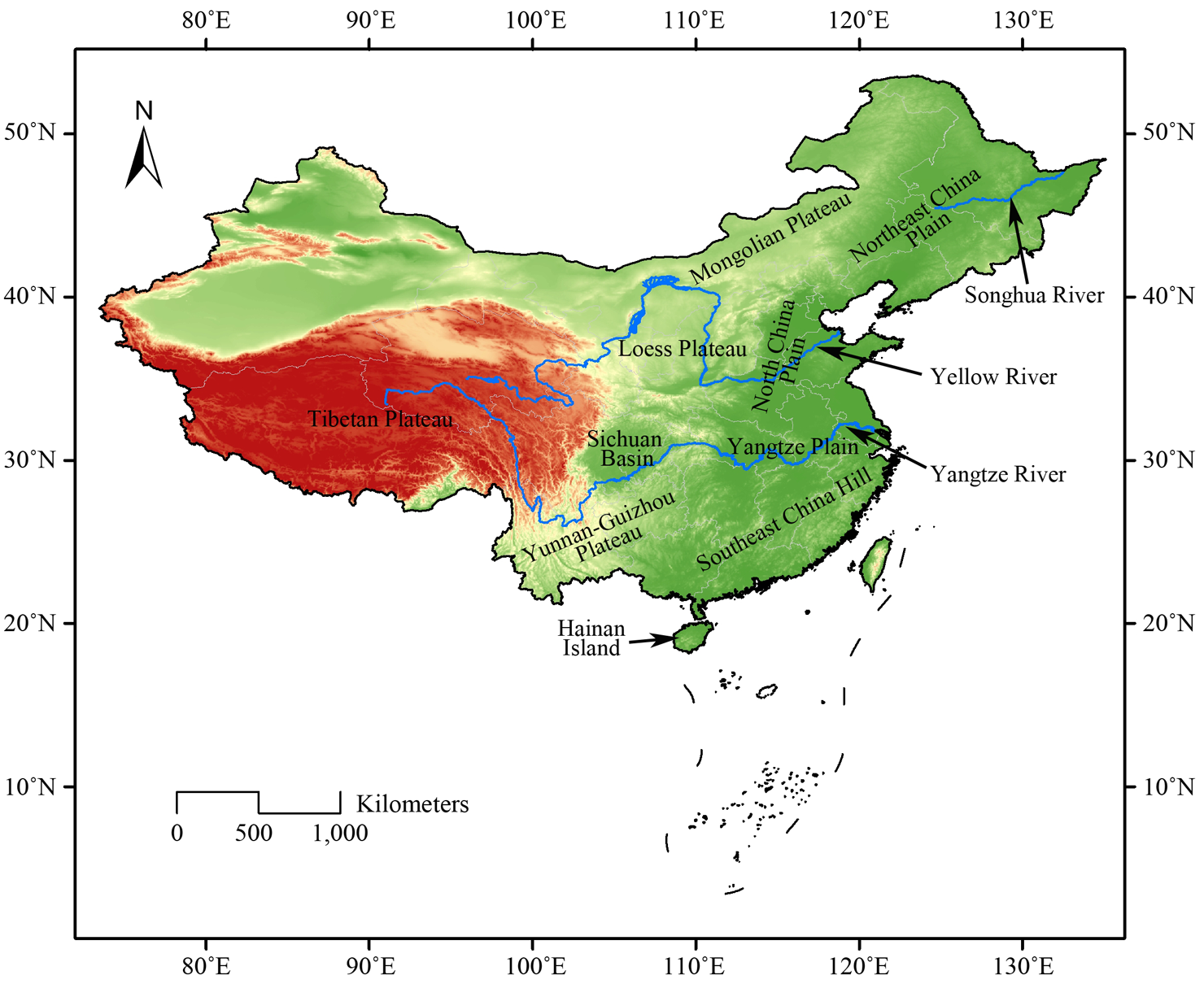

Supplement: Figure S1 — Regions included in the present study. (TIF) [file pone.0111587.s001.tif]

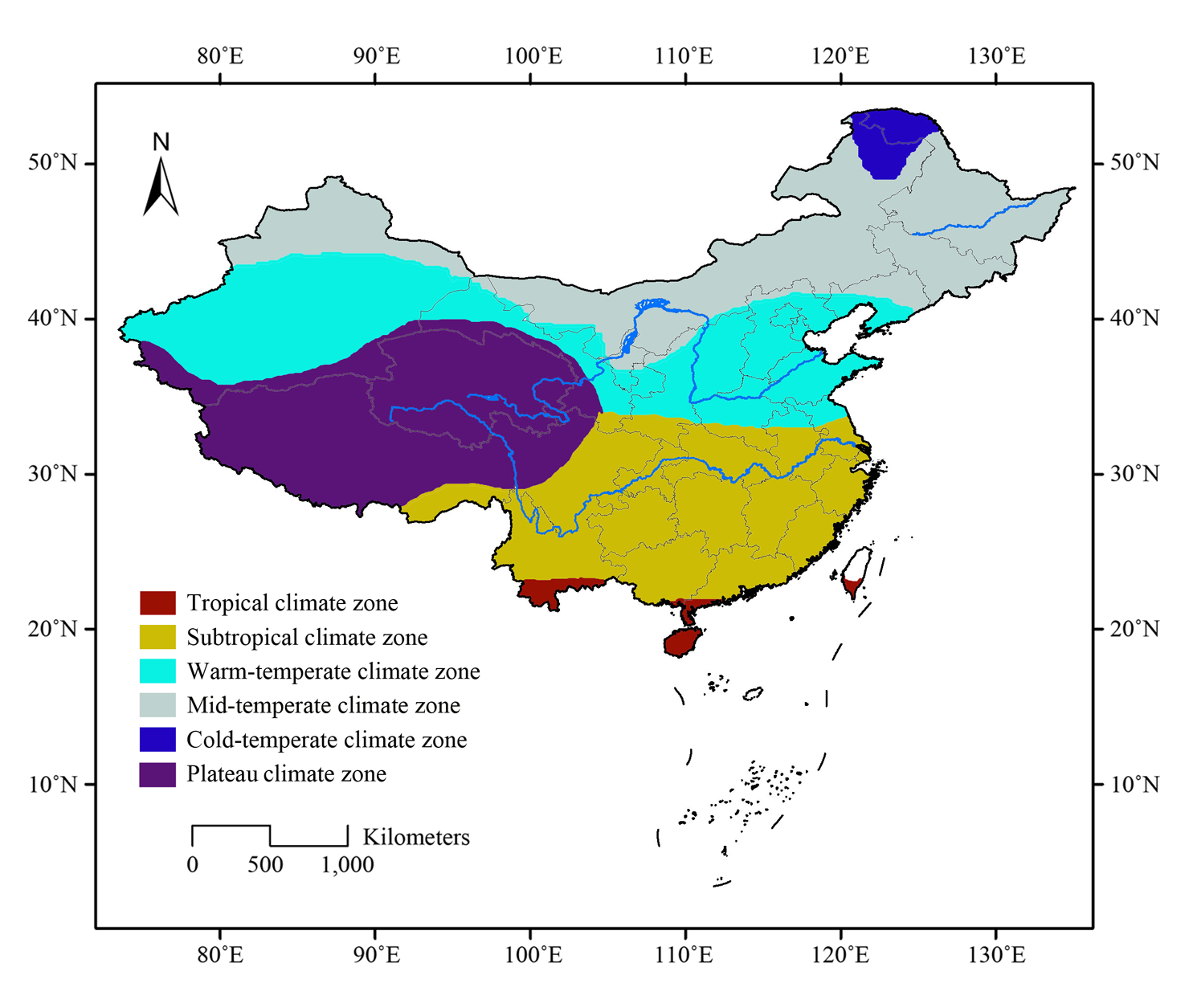

Supplement: Figure S2 — Climate zones in China. (TIF) [file pone.0111587.s002.tif]

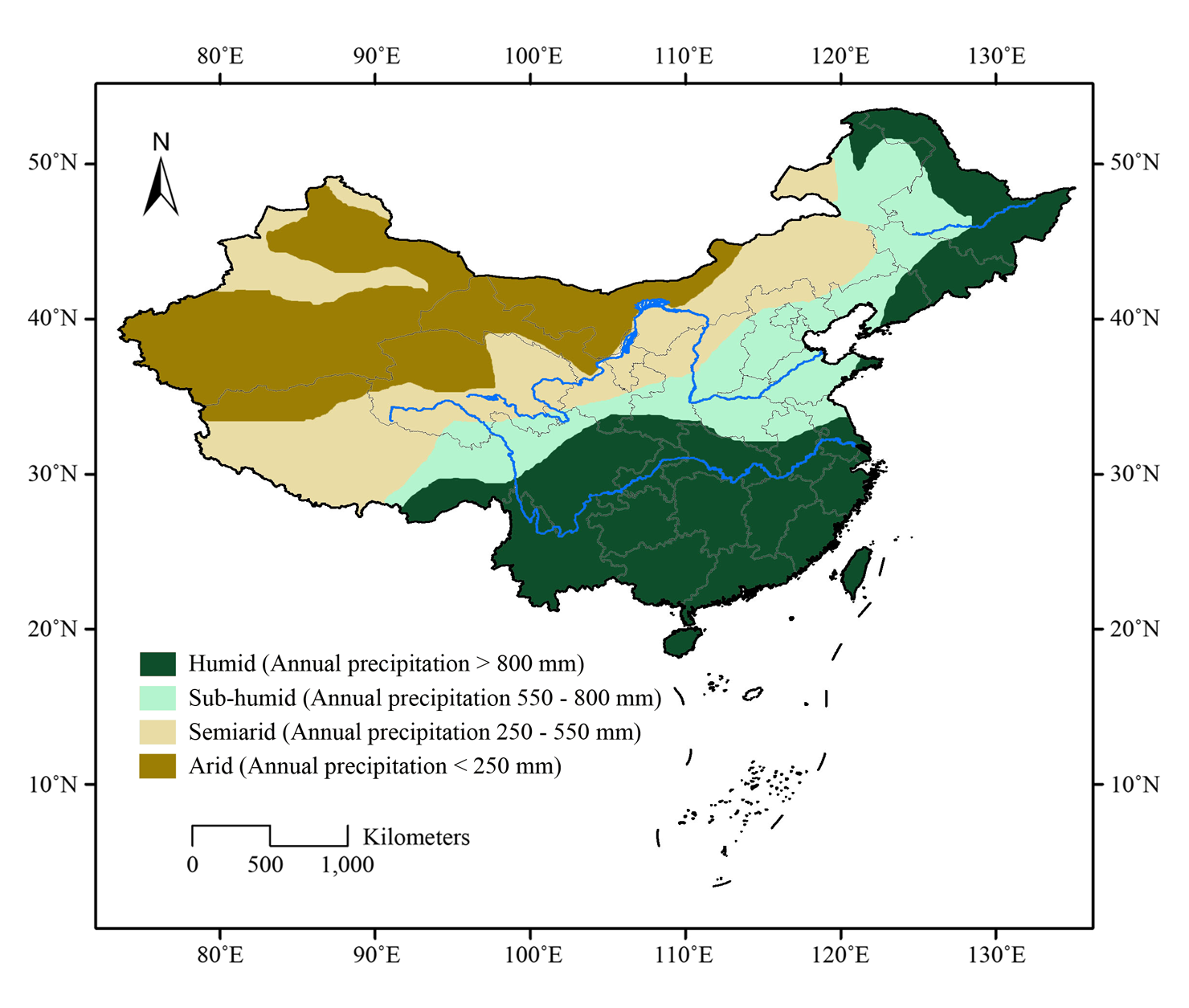

Supplement: Figure S3 — Distribution of humid and arid areas in China. (TIF) [file pone.0111587.s003.tif]
